# Preferences for the provision of whole genome sequencing services among young adults

**DOI:** 10.1371/journal.pone.0174131

**Published:** 2017-03-23

**Authors:** Christopher H. Wade, Kailyn R. Elliott

**Affiliations:** 1 School of Nursing & Health Studies, University of Washington Bothell, Bothell, Washington, United States of America; 2 School of Medicine, University of Washington, Seattle, Washington, United States of America; TNO, NETHERLANDS

## Abstract

**Objectives:**

As whole genome sequencing (WGS) becomes increasingly available, clinicians will be faced with conveying complex information to individuals at different stages in life. The purpose of this study is to characterize the views of young adults toward obtaining WGS, learning different types of genomic information, and having choice about which results are disclosed.

**Methods:**

A mixed-methods descriptive study was conducted with a diverse group of 18 and 19-years-olds (N = 145). Participants watched an informational video about WGS and then completed an online survey.

**Results:**

Participants held a positive attitude toward obtaining WGS and learning about a range of health conditions and traits. Increased interest in learning WGS information was significantly associated with anticipated capacity to handle the emotional consequences if a serious risk was found (*β* = 0.13, *P* = .04). Young adults wanted the ability to choose what types of genomic risk information would be returned and expressed decreased willingness to undergo WGS if clinicians made these decisions (t(138) = -7.14, *P* <.01). Qualitative analysis showed that young adults emphasized procedural factors in WGS decision-making and that perceived health benefits of WGS had a substantial role in testing preferences and anticipated usage of WGS results.

**Conclusions:**

Clinicians are likely to encounter enthusiasm for obtaining WGS results among young adults and may need to develop strategies for ensuring that this preference is adequately informed.

## Introduction

As WGS becomes increasingly available in standard clinical care, there has been interest in expanding the use of WGS beyond diagnostic purposes to prevent disease and facilitate early treatment. This raises the question of when in a patients' lifespan WGS services should be provided as part of standard practice [1]. Although some have suggested that WGS could be integrated into pediatric care [2,3], the approach that is currently recommended is to postpone offering WGS services until a patient reaches the age of majority (e.g. 18-years-old in most countries). So, in this model, the transition into young adulthood and legal independence may be accompanied by a decision about whether to undergo WGS.

The perspective that WGS should be postponed until adulthood is supported by most existing clinical guidelines for genetic testing [4,5]. For example, in the United States a 2013 American College of Medical Genetics (ACMG) report suggested that minors should not be offered routine carrier testing and that predictive testing should be deferred unless it would cause substantial psychosocial distress [5]. This argument is justified in part by citing the limited clinical utility of childhood predictive testing for adult conditions and the potential impact of risk information on children and their families. Because WGS can reveal carrier status, predictive genetic risks, and a wide range of other health risks that vary in severity and clinical utility, some see these results as typically being more appropriate for adults [6,7].

The views of young adults toward WGS may be influenced by preferences and needs that are distinct from older adults [8]. In particular, their personalities are still evolving and they express less aversion to risks [9]. From a life course perspective, young adults have also been shown to integrate the meaning of genetic risks differently than older individuals with respect to existential factors, social relationships, and behaviors [10,11]. Limited research has been conducted with young adults on their preferences for WGS, although studies on WGS with graduate students in health-care professions [12,13] and direct-to-consumer genetic testing with undergraduates suggest that they may have considerable interest in obtaining testing [14,15]. Additionally, studies that look across adult age-ranges find that adults generally are favorably inclined toward obtaining WGS risk information [16,17].

A significant clinical dilemma concerns the degree of choice patients should have in determining what types of WGS information is disclosed. Older adults tend to prefer the option of choice in genetic testing and some evidence indicates that they would deliberately select among different types of genetic information if given the opportunity [17–19]. Genetics professionals also express support for patient choice among types of WGS information [20,21]. However, the wide scope of information provided by WGS presents a major challenge for achieving fully informed decision-making and too many options may distort decision-making processes [22]. Some tools are being developed to assist in the decision-making [23,24], but the potentially serious consequences of withholding risk information makes complete reliance on the validity of patient choice controversial. For example, this concern underlies mandatory newborn screening programs [25] and recent ACMG guidelines for WGS that recommend disclosing significant unanticipated genetic risks regardless of patient preferences [26].

Understanding the perspectives and preferences of young adults toward WGS is necessary for clinicians to effectively support them in test decision-making, and this will be particularly important if WGS is postponed until the age of majority. Furthermore, the complexity of these conversations may increase if WGS is offered through a commercial or public health screening approach, where the depth of counseling prior to testing and genomic information available may vary. Therefore, the objectives of this study is to describe the attitudes of young adults toward obtaining WGS services, receiving different types of WGS information, and having choice about which types of WGS results are disclosed.

## Methods

### Study design

This descriptive mixed methods study was conducted at the University of Washington Bothell in 2014. The University of Washington Human Subjects Division assessed this study and determined that it was exempt from review (Application #46507). A sample of 18 and 19-year-old undergraduates were recruited in person during visits to 20 courses that target first-year students. Students were introduced to the study, informed of the incentives (a $5 gift card incentive and entry into a raffle for two iPads (Apple, Cupertino, CA)), and then asked to provide contact information if they wanted to participate. Students who expressed interest in the study were emailed a link to a confidential online survey using the University of Washington Catalyst WebQ system (https://catalyst.uw.edu). After reading an introduction to the study, students included in the study selected an option indicating their consent to participate.

Survey participants viewed a 10 minute animated video called “Whole Genome Sequencing and You” (https://goo.gl/HV8ezJ), which was designed to be accessible to the general public and teach the basics of genetics, explain WGS, and describe the types of information that WGS could provide (e.g. genetic disease risk, carrier status, susceptibility to common diseases, pharmacogenomic information, traits, ancestral background, and genetic variants of unknown significance) [27]. Participants were then asked to complete a survey. Institutional records were used to generate aggregate demographic data about participants and non-participants in the classes where students were recruited.

### Survey and measures

The survey was designed to collect data about sociodemographic factors and attitudes toward WGS. During development it underwent review and revision based on feedback from a group of interdisciplinary experts and a pilot survey with 20 undergraduate students. The survey items and sources can be obtained online (S1 Document and S1 Table). Variables and anchors can be found in Tables 1 and 2.

**Table 1:** Sociodemographic characteristics.

Characteristic	Aggregate Institutional Data		Respondents' Self-reported Characteristics (n = 145) %^a^^b^(n)
	Non-Respondents (n = 141) %^a^ (n)	Respondents (n = 145) % ^a^ (n)	
**Age**			
18	43 (60)	44 (64)	48 (69)
19	58 (81)	56 (81)	52 (75)
Not indicated	.	.	1 (1)
**Gender**			
Male	45 (63)	37 (54)	37 (54)
Female	55 (78)	63 (91)	63 (91)
**Ethnicity**			
Hispanic or Latino	11 (16)	13 (19)	13 (19)
Not Hispanic or Latino	89 (125)	87 (126)	87 (125)
Not indicated	.	.	1 (1)
**Race**			
African American	9 (13)	6 (9)	6 (9)
Alaskan Native or American Indian	0 (0)	0 (0)	0 (0)
Asian or Asian American	32 (45)	41 (60)	42 (61)
White	38 (54)	30 (44)	32 (46)
Native Hawaiian or Pacific Islander	3 (4)	0 (0)	1 (2)
Two or more races	8 (11)	10 (15)	9 (13)
Other	.	.	6 (8)
Not indicated	10 (14)	11 (17)	4 (6)*
**Parents' Annual Household Income**			
≤$25,000	.	.	10 (14)
$25,001-$50,000	.	.	27 (39)
$50,001-$75,000	.	.	14 (20)
>$75,000	.	.	30 (44)
Not indicated	.	.	19 (28)
**First Generation College Student**			
Yes	52 (73)	53 (76)	.
No	48 (67)	47 (67)	.
Not indicated	1 (1)	1 (2)	.

^a^ Percentages may total to more than 100% because of rounding.

^b^ Asterisk indicates a significant difference between respondents' self-reported characteristics and aggregate institutional data (p ≤ 0.05)

**Table 2:** Participant traits and attitudes.

Variable	Response N = 145, Mean (+/- S.D.)
Self-assessed health status (1 = Excellent, 5 = Poor)	2.49 (.94)
Importance of learning impact of behaviors on health (1 = Not at all important, 7 = Very important)	5.61 (1.33)
Importance of learning impact of genes on health (1 = Not at all important, 7 = Very important)	5.29 (1.41)
Motivation to take action to improve health (1 = Not at all motivated, 7 = Very motivated)	4.54 (1.26)
Knowing WGS results would lead to change in behavior (1 = Not at all likely, 7 = Very likely)	3.66 (.82)
Able to handle emotions if WGS revealed risk for a serious health condition (1 = No, I would not, 7 = Yes, I would)	4.86 (1.56)
***Interest in learning WGS information about conditions and traits (1 = Not at all interested*, *7 = Very interested)***	
Pharmacogenomic information	5.92 (1.23)
Predictive information about preventable health conditions	5.88 (1.22)
Slight or moderate susceptibility to health conditions	5.77 (1.31)
Carrier status	5.70 (1.57)
Ancestry	5.59 (1.69)
Non-health related traits	5.20 (1.77)
Predictive information about non-preventable health conditions	4.84 (1.69)
*Average interest across the types of WGS information*	*5*.*60 (*.*98)*
***Decisional Balance (1 = Strongly disagree*, *7 = Strongly agree)***	
Benefits of WGS (Average of 5 items)	5.44 (1.04)
Risks of WGS (Average of 5 items)	3.11 (.98)
*Decisional balance ratio (Risks/Benefits)*	.*60 (*.*29)*
**Willingness (1 = Not at all willing, 7 = Very willing)**	
Willingness to have WGS in general	5.28 (1.48)
Willingness to have WGS when a clinician decides which risk information is provided	4.23 (1.73)
*Willingness ratio (Willingness when clinician decides/Willingness in general)*	.*84 (*.*39)*
Importance of being able to choose what types of WGS information is reported (1 = Not at all important, 7 = Very important)	5.92 (1.38)

#### Participant characteristics

Validated measures were used or adapted to assess age, gender, parent's household income, and current health status. Measures of first generation student status, ethnicity, and race were obtained from the University of Washington Bothell institutional data collection tool.

#### Attitudes toward WGS

A scale measuring decisional balance was adapted from another study [28] for the assessment of attitudes toward WGS. The decisional balance ratio was generated by dividing responses to five items about the risks of WGS (e.g. “It might tell me something I don’t want to know”) (α = .86) by the responses to five items about the benefits of WGS (e.g. “It would help me to pay more attention to my health”) (α = .63). Additional information relevant to attitudes was gathered by using or adapting validated measures (willingness to get WGS, importance of behavior, importance of genes) and using items developed for this study (motivation to improve health, knowing a person with a genetic disorder, ability to handle emotions, and willingness to pay for WGS).

#### Preferences for different types of WGS information

Interest in WGS was assessed for seven categories of information that could be provided by WGS. A definition and example was provided for each category, and a likert scale was used to assess interest in learning each type of information. (e.g. “Information Type B: Genomic information could show that a health condition is very likely to develop. Unfortunately, you can not take action to decrease your chances of the condition occurring. *Example*: People with a certain genetic risk are very likely to develop Alzheimer’s disease, which is a fatal neurological condition that typically occurs later in life. There is no effective way to prevent this disease from occurring. Would you want to know about this kind of information?”). A measure of average interest in learning genomic information was generated by taking the mean of the seven types of genomic information (α = .78). To assess interest using a dichotomous measure (yes/no), participants were also asked "Imagine that you had whole genome sequencing. Would you want to know if you had increased chances of developing the following health conditions or traits?" This was followed by seventeen options, which are listed in the results section.

#### Choice of WGS information disclosed

The willingness ratio measures the difference between interest in getting WGS in general (“How willing would you be to have your whole genome sequenced?”) and interest in getting WGS in a scenario where a clinician selects the information to be reported (e.g. “Imagine your healthcare provider decided what information to tell you about your whole genome sequence. Assume your provider did their best to only tell you information that he or she thought could improve your health. You do not get to decide what types of information you are told. In this scenario, how willing would you be to undergo whole genome sequencing?”). To avoid bias, the two questions were asked at different points in the survey. The willingness ratio was calculated by dividing scores for the restricted choice scenario by the scores for whole genome sequencing in general. Additional questions designed for the study assessed the importance participants placed on being able to choose the WGS information disclosed and a measure that assessed the level of detail they desired for this choice.

#### Qualitative questions

Two open-ended questions were asked at the end of the survey. The first addressed factors in WGS uptake decision-making: "Imagine you had to decide whether or not to get whole genome sequencing. What factors would be most important to your decision." The second addressed how they would use WGS information: "Imagine you got whole genome sequencing. How do you think that you would use the information that you learned?"

### Analysis

Study data was cleaned and analyzed in SPSS version 23 (IBM, Armonk, NY). Frequencies, measures of central tendency, and data variability were calculated. A Fisher’s exact test was used to detect differences between the sociodemographic characteristics of non-responders and responders. A paired t-test was used when it was necessary to assess the difference between means. Pearson’s product-moment correlation or Spearman’s rank correlation coefficients were used as was appropriate to the data type. Multivariate linear regression was conducted by simultaneous entry; variables selected for inclusion had a bivariate correlation of *p* ≤ .20 with dependent variables, while variables that overlapped with the dependent variable were excluded. All p-values refer to two-tailed analysis and p-values ≤ .05 were reported as significant. The sample provides a power of over 0.8 to detect effect sizes of 0.35 for tests of difference, population correlations of 0.25, and population R^2^ of 0.10 for regression analysis.

The open-ended qualitative questions were analyzed using an inductive content analysis strategy [29]. Initial codes were developed independently by both authors. A coding schema was then synthesized collaboratively and applied to the text using the qualitative analysis software Dedoose v.7.0.23 (Dedoose, Hermosa Beach, CA). The coding procedures and definitions were refined by coding over half of the responses in a training round. Once procedures were finalized, both authors coded the entire dataset independently. Intercoder reliability was then evaluated using Cohen's kappa (values over 0.8 have been described as indicating excellent agreement [30]). Code applications were finalized by resolving coding differences through consensus.

## Results

### Participant characteristics

Most qualified young adults expressed interest in the study (93%, 286/307) and were emailed a link to the survey. A total of 145 qualified participants completed the survey, resulting in a 51% response rate among students who received a survey. Study sociodemographic characteristics can be found in Table 1. To assess the possibility of bias, aggregate institutional data was used to compare the characteristics of respondents to contacted non-respondents. No significant differences were identified. Participants' self-reported characteristics collected in the survey were also similar to the responders’ characteristics from aggregate institutional data (with the exception that more indicated their race). Since the data on characteristics obtained through the survey is individual rather than aggregate, only the self-reported data is used in the following analysis.

Survey responses provided insight into the outlook of this sample of young adults (Table 2). Most were in good or very good health and expressed a moderate level of motivation to take actions that would improve their health. Overall, they endorsed both behaviors and genetics as being important for health, but their emphasis on behavioral contributions was significantly higher (t(144) = 2.6, p = .01). Approximately a third of the students (35%) reported knowing someone who had a genetic disorder that influenced their health.

### Attitudes toward WGS

Young adults typically expressed willingness to obtain WGS (Table 2). Additionally, they endorsed the idea that WGS would help them change their health behaviors and expressed moderate confidence in their ability to handle their emotions if they learned about risk for a serious health condition (Table 2). On the whole, participants were willing to pay a small amount for WGS (median $1-$50), but many were unwilling to pay anything (34%). Young adults had a favorable overall attitude toward WGS as measured by the decisional balance ratio, where participants endorsing five positively worded items about WGS (e.g. important, has prevention potential, would put mind at ease) more favorably than five negatively worded items (e.g. useless, invasion of privacy, receive unwanted information) (Table 2).

### Preferences for obtaining different types of genomic information

Study participants read definitions and examples of different types of genomic information and then evaluated their interest in obtaining that information for themselves (Table 2). Overall, the 18 and 19-year-olds expressed interest in receiving all types of WGS information, although the magnitude varied by information type. Correlations were investigated between all types of genomic information and all sociodemographic characteristics (S2 Table) and attitudinal factors (S3 Table) and variables with significant associations are reported in the supplementary materials. A composite average measure of interest in learning genomic information was generated from the seven types of information (α = .78) and factors associated with it were identified (Table 3). In a regression model assessing correlated factors, the only factor that remained significantly associated with higher average interest was a greater confidence in ones' ability to handle the emotional consequences if WGS revealed a serious risk (Table 4).

**Table 3:** Factors correlated with average interest in learning genomic information and the willingness ratio.

Variable ^a^	Average Interest in Learning Genomic Information, *r* (*P*)^b^	Willingness Ratio, *r* (*P*)^b^
Age	-.06 (.46)	-.17 (.05)*
Parental income	.17 (.07)	-.01 (.95)
Hispanic or Latino	.13 (.12)	-.09 (.28)
Asian or Asian American	-.06 (.50)	.12 (.18)
Caucasian or White	.13 (.14)	-.15 (.08)
Native Hawaiian or other Pacific Islander	.16 (.07)	.11 (.20)
Two or more races	.18 (.05)*	-.07 (.40)
Importance of learning impact of behaviors on health	.15 (.09)	.07 (.39)
Importance of learning impact of genes on health	.42 (<.01)*	.00 (.97)
Motivation to take action to improve health	.16 (.07)	.05 (.58)
Know a person with a genetic disorder	.06 (.52)	.24 (<.01)*
Knowing WGS results would lead to change in behavior	.17 (.05)*	-.00 (.97)
Ability to handle emotions if a risk was found for a serious health condition	.30 (<.01)*	-.24 (<.01)*
Decisional balance ratio	-.50 (<.01)*	.12 (.15)
Importance of choice	.23 (<.01)*	-.36 (<.01)*

^a^ Factors selected for inclusion in the table had an association of *P* ≤.2 with at least one dependent variable.

^b^ Asterisk highlights correlations of *P* ≤.05.

**Table 4:** Regression analysis of factors associated with average interest in learning genomic information and the willingness ratio.

Variable	β	SE of β	*t*	*P*^a^
**Average Interest in Learning Genomic Information (R**^**2**^ **= .17)**				
Parental income	.14	.09	1.54	.13
Hispanic or Latino	.36	.27	1.31	.19
Caucasian or White	-.02	.20	-.08	.93
Pacific Islander or Native Hawaiian	1.10	.57	1.94	.06
Two or more races	.31	.30	1.06	.29
Motivation to improve health behaviors	.08	.07	.99	.33
Knowing WGS results would lead to a change in.behavior	.11	.11	.95	.34
Ability to handle emotions if a risk was found for a serious health condition	.13	.06	2.09	.04*
Importance of choice	.9	.06	1.34	.18
**Willingness ratio (R**^**2**^ **= .17)**				
Age	-.15	.06	-2.60	.01*
Asian or Asian American	.03	.08	.37	.71
Caucasian or White	-.10	.08	-1.31	.19
Pacific Islander or Native Hawaiian	.16	.24	.66	.51
Know a person with a genetic disorder	.13	.06	2.03	.04*
Ability to handle emotions if a risk was found for a serious health condition	-.03	.02	-1.27	.21

^**a**^ Asterisk indicates p ≤.05.

When asked to provide a dichotomous answer (Yes/No) about whether they would want to learn their WGS results for 17 specific conditions and traits, the majority (52%) of participants selected every available option. From highest to lowest, the percentage of affirmative responses were: Breast cancer (92%); Lung cancer (92%); Long QT syndrome (92%); Heart disease (91%); Diabetes (89%); Responding well to a medication (89%); Risk of having a child with cystic fibrosis (86%); Alzheimer’s disease (84%); Depression (84%); Having ancestors that lived in a specific location (84%); Memory traits (84%); Obesity (82%); Athletic ability (79%); Appearance traits (79%); Flu virus infection risk (78%); Prostate cancer (76%); and Alcoholism (76%).

### Importance of choice about the disclosure of different types of WGS information

Respondents typically expressed a strong interest in having a choice about the types of genomic information they would receive from WGS (Table 2). The importance of choice was also assessed by comparing overall willingness to undergo WGS with willingness to undergo WGS in a restricted choice scenario; the two questions were asked at different points in the survey to minimize bias (Table 2). Specifically, respondents were presented with a scenario in which a clinician only provided information judged to have clinical utility and the patient could not decide what information they were given. Willingness to have WGS significantly decreased when patient choice was restricted (t(138) = -7.14, *P* <.01), although the young adults remained moderately receptive to testing in this scenario. A ratio that measured this change in willingness was created by dividing willingness to have WGS in the restricted scenario by overall willingness to have WGS. Factors correlated with the willingness ratio were identified (Table 3). Regression analysis found that the decrease in willingness to have WGS when choice was restricted was significantly more pronounced among participants of older age and those who knew someone with a genetic disorder (Table 4).

The young adults were also asked what degree of control they wanted when choosing the WGS information that would be reported. The options ranged from no control (e.g. clinicians make all decisions) to controlling the disclosure of each specific condition or trait (e.g. diabetes, depression, athletic ability, etc.). The median preference was to be able to decide the general categories of information returned (e.g. predictive and preventable, pharmacogenomics, etc.).

### Factors influencing decisions about whether to undergo WGS

In an open-ended question, participants were asked to describe the factors which they believed would impact their decision-making with respect to seeking out WGS. Most participants (n = 140) answered the question, with an average response length of 129 characters. Content analysis revealed that the young adults focused on factors that fit into four thematic categories (Table 5). The first category addressed the processes for WGS provision, with the ability to choose what WGS information they would receive and the financial burden of WGS being common themes. For example, one participant stated: "The factors that would be most important would be to be able to decide what information I receive and who is given my information, as well as an assurance/confirmation that any genetic material remaining after the sequencing is completed is destroyed" (#106). Somewhat less frequently participants mentioned a desire to know about how the privacy of their information would be ensured and wanted control over how their genetic information would be used by other parties. Second, the young adults indicated that a major factor in their decision-making would be the anticipated health benefits of obtaining WGS. They often described this factor in broad terms, but sometimes specified the ability to prevent the onset of future diseases. In the words of one young adult: "The factors that would be important are if there are any treatments that would help me improve my chances of not developing health conditions and how it would help doctors improve any future treatments" (#99). Also, some participants suggested that their desire to undergo WGS would be dependent on perceived need based on their health status when they were making their decision. Third, a subset of respondents described their interest in WGS as being connected to familial and social ties. This took the form of shared familial risks, both to current family members and to future children, as well as in a desire to be able to learn about ancestry. This is illustrated in the statement: "My dad is adopted, and we don't know what health risks/factors that come along with his genetics. Knowing what they were would make or break my decision." (#71). Others speculated that their loved ones' preferences or probable responses to risk would play a role in whether they pursued WGS. In a fourth thematic category, participants described emotional and cognitive considerations for WGS services. A subset described how their perceived ability to cope with learning genetic risks might influence their decisions about seeking WGS, while others mentioned the need to consider how much their life would change overall. One participant explained that a factor in their decisions was "Whether or not I would be able to handle finding out that I was going to [get] Alzheimers when I'm older, or cancer, because although I'm interested in finding out what I need to do [to] prevent myself from having certain ailments, I don't know if I can handle finding out that I'm going to have a terminal illness" (#134).

**Table 5:** Reported factors that would influence decisions to obtain WGS.

Themes	n (%, κ^a^) (n = 140)	Representative Quotes
**Process Factors**		
Selection of Information	33 (24, .87)	*"What all info I will be receiving and making sure I can have the option of choosing to receive all the information*.*" (#125)*
Cost	33 (24, 1.00)	*"The cost*. *To me this has to be something affordable by anybody*. *Even someone in college who has almost nothing*.*" (#94)*
Privacy & confidentiality	12 (9, .90)	*"I would want to know that my privacy is guaranteed*.*" (#53)*
Control of information use	7 (5, 1.00)	*"How my genetic information will be used by researchers and people/institutions that I do not know*.*" (#115)*
Accuracy	5 (4, 1.00)	*"*.* *.* *. *If the information would be highly accurate*.*" (#26)*
Timing of process	5 (4, 1.00)	*"How long it would take to get the results*.*" (#127)*
Invasiveness	3 (2, .85)	*"If it will hurt me physically*.* *.* *.*" (#63)*
**Health Utility Factors**		
Overall health	35 (25, .98)	*"*.* *.* *.*I know that if I'm contemplating getting genome sequencing it's because I want to know more about myself medically*.*" (#122)*
Potential for prevention	26 (19, .91)	*"*.* *.* *.*factors that would be most important to my decision is the results of my health conditions and what can be done to improve it*.*" (#57)*
Current health status	5 (4, 1.00)	*"*.* *.* *. *my current health at the time*.*" (#72)*
**Social Factors**		
Family health history	8 (6, .87)	*"Knowing that a family member has a genetic disease would increase my chances in getting whole genome sequencing*.*" (#80)*
Ancestry	8 (6, 1.00)	*"Some factors would be my curiosity for what my ancestral heritage is*.*" (#87)*
Reproduction & children	8 (6, .93)	*"Am I considering having children*? *If it turns out bad*, *I might consider adoption*.*" (#3)*
Attitudes of loved ones	6 (4, .85)	*"I believe important factors would be how my parents would react*. *I know I would want to tell them at least some of the information and then they would be curious to know more and that may mean me lying to them to give them positive results so they won't have to worry*.*" (#31)*
**Emotional & Cognitive Factors**		
Ability to cope	14 (10, 1.00)	*"The emotional impact would be very important because for example if I learned that I would have a heart problem*, *I would live my life waiting for a heart attack to happen*.*" (#95)*
Impact on life	6 (4, .91)	*"How it would change my life*.*" (#82)*
Uncertain	4 (3, .85)	*"I do not know*.*" (#32)*

^a^ Cohen's kappa scores evaluating intercoder reliability were calculated after individual coding and prior to reaching a consensus on code application.

### Anticipated usage of WGS results

The young adults were asked to explain, in their own words, how they would use WGS results if they were tested. The majority of participants responded (n = 139) with an average passage length of 117 characters. Although a few participants stated no anticipated use (n = 3) or were unsure (n = 4), content analysis revealed that the majority of responses fell into three thematic categories for using WGS (Table 6). In the first and most prominent category, many young adults expected that WGS information would be used to improve their health and prevent disease. When specifics were mentioned, this was most frequently described as being accomplished through primary prevention (e.g. improved health habits like eating behaviors), but they also addressed clinical applications (e.g. directing medical treatment, improved pharmaceutical usage, and discussions with providers). For example, one participant stated that "I would use the information to prevent any preventable diseases. I would also be more prepared if I get a disease in the future. Most of all, it would give me motivation to live healthier" (#97). The second thematic category encompassed cognitive uses. This commonly took the form of broad statements suggesting that WGS would help guide their lives in a positive direction or assist in learning about themselves and being more aware. A subset also expressed that WGS would allow them to be prepared for getting a health condition, particularly in the context of non-preventable diseases. A third thematic category described social applications of WGS results. Several participants expressed a desire to use their experience with WGS to help others by raising awareness or teaching about health risks. Most other themes were contextualized in shared genetic risks within families, both as a way to identify relatives at greater risk and as a means to improve future reproductive decisions or child rearing. One participant illustrated this by saying they would use the information with "my friends and family if they have the similar conditions. I will then use it to know if it is dangerous to have a child that could be a carrier to my health conditions" (#125).

**Table 6:** Anticipated uses of WGS information.

Themes	n (%, κ^a^) (n = 139)	Representative Quotes
**Health Uses**		
General health	72 (52, .99)	*" The information would be beneficial because I would be able to guide my health in a positive direction and reduc[e] my chances of having a disease*.*" (#126)*
Change health habits	31 (23, .98)	*"I would try to conduct healthier eating and exercise habits in order to try to prevent or lessen the health issue that I may get*.*" (#62)*
Change pharmaceutical usage	8 (6, .85)	*"I would definitely use the information if it tells me what medicine is best for me to take*.*" (#38)*
Guide medical treatment	4 (3, 1.00)	*"*.* *.* *.*it would help doctors decide which treatment is best for me*.*" (#41)*
Talk to a clinician	3 (2, 1.00)	*"I would try to prevent the preventable disorders by talking to the doctors about how to minimize risk*.*" (#65)*
**Cognitive Uses**		
Guide life	24 (17, .97)	*"It would change me for the better and lead me toward "the right path*.*" (#113)*
Inspire learning or awareness	22 (16, .97)	*"Live with a new perspective on life and see things through a whole new lens*.*" (#2)*
Prepare self for disease	6 (4, .92)	*"If I found out I had a serious disease it would give me time to get in the right mind set and spend the time I had left with those I love*.*" (#110)*
**Social Uses**		
Help or inform others	13 (9, .96)	*" I think I would try to help others with the same issues as me*.*" (#27)*
Reproductive decision-making	7 (5, .92)	*"It would also help me decide whether I should adopt a child or have children myself*.*" (#39)*
Family risks	7 (5, .93)	*"I would probably use it to help my family*. *Maybe they would have the same issues as me and can also get help from it*.*" (#46)*
Ancestry	6 (4, 1.00)	*"I would use the information to*.* *.* *. *find out my actual roots because from both sides of our family are very distant from us*.*" (#91)*
Guide health of children	3 (2, .85)	*"It would allow me to*.* *.* *. *prepare my kids for the traits that could pass on to them*.*" (#111)*

^a^ Cohen's kappa scores evaluating intercoder reliability were calculated after individual coding and prior to reaching a consensus on code application.

## Discussion

As the accessibility of WGS continues to improve, clinicians and policymakers will need to clarify expectations for who can obtain these services and what genomic information will be reported. The findings of this study demonstrated that young adults ages 18–19 have considerable interest in obtaining WGS and were positively inclined toward receiving all types of genomic information examined in the study. Although the young adults had a desire for choice among different types of information, a majority of participants did not appear to exercise that option when selecting the genomic information they would want to be reported.

The positive attitudes expressed by study participants toward genomic information are consistent with similar research among young adults and adults in general [12–17]. Systematic reviews that look at genetic testing across a range of disease types have also revealed this trend [31]. In this study, participants reported a favorable decisional balance, high average interest in all WGS information types, and willingness to obtain WGS. These results have implications for healthcare providers because they suggest that clinicians may encounter considerable enthusiasm when initially discussing WGS with young adults. In the face of such optimism, clinicians may have difficulty ensuring that patients comprehend key points that are crucial to informed decision-making about WGS; in particular the fact that a small portion of individuals will learn results that are severe and potentially life changing. Concerns about young adults' comprehension of WGS are significant, even medical students enrolled in a course on genomics who had received actual WGS results were found to have significant gaps in understanding [13]. There is reason to believe that efforts to provide genomics education may succeed in tempering enthusiasm and supporting informed decision-making, since interest in genetic testing tends to decrease as more information is provided [32]. It is also notable that participants in this study who had more concern about their emotional coping skills were less interested in learning WGS, suggesting that young adults may appropriately self-select when opting for testing. A separate analysis of how these participants viewed WGS for newborn screening also indicated that a subset of participants had considerable concerns about their ability to cope with severe genomic risks [33]. The importance of emotional factors emphasizes that clinicians will have to prepare to help young adults understand their emotional needs as well as simply providing factual information about genomics.

In this study, both quantitative and qualitative data indicated that participants have high expectations for the ability of WGS to improve their health, despite the fact that the clinical utility of WGS as a population screening tool remains unclear. Also, the potential uses for which participants expressed enthusiasm, such as personal traits, extend far beyond the domain currently addressed in clinical applications of WGS. There is little evidence to support young adults' belief that WGS will inspire primary prevention through behavior change. In fact, systematic reviews have not revealed beneficial behavior change following testing for genetic risks [34] except in the context of high risk, effective prevention measures, and a strong family history [35]. The perceived actionability of findings did appear to be a consideration for the young adults; genomic information types where clinical or preventive options were indicated generated greater interest. Given the vast amount of information provided by WGS, clinicians will need to narrow the scope of what is addressed with young adults in their care. The emphasis that young adults place on actionability suggest that they may be receptive to strategies that prioritize information based on tiered risks and prevention potential, even if they still want access to other findings [36,37]. Additionally, given that the participants were typically unwilling to pay for WGS, emphasis on the cost of analysis may contextualize the importance of focusing on findings with significant health risks.

The complexity of WGS results underscores the need to develop systems for including patient preferences in the return of results [23]. Research with children [19], adults [38], and genetics professionals [20] indicate a strong endorsement of providing options, as did the participants in this study. The young adults stated that choice was very important to them and expressed lower willingness to be tested if clinicians made all choices about what results were reported. The level of control that they wanted over the information received varied, but they typically wanted to have choice about the general types of genomic information (e.g. pharmacogenomic, predictive and preventable, etc.). Despite the fact that they expressed a desire for choice and evaluated categories of WGS information types differently, the majority of young adults selected every option among 17 types of conditions and traits that ranged from severe (e.g. Alzheimer’s disease) to factors with few clinical implication (e.g. appearance traits). Therefore, young adults may not be fully considering the option to decline results. This is important because patients are likely to make such decisions by developing a “gist” understanding of complex information [39] and in this case it may be acting in opposition to careful informed consent. It could be that these findings simply reflect a decreased engagement in details due to the hypothetical nature of the survey. It is also plausible that many younger adults would simply want all available information, a perspective that has been observed in other adults [19,38]. Given that WGS produces an abundance of incidental findings, the findings of this study suggest that many patients would prefer that clinicians offer a wide range of information types, even ones that are not clinically actionable (e.g. traits). This emphasizes the importance of clarifying and justifying when clinicians will withhold WGS information.

In interpreting the findings of this study, it is worth noting several strengths and limitations. The study had a diverse sample with no evidence of selection bias and a response rate that is respectable for an online survey. However, it is well known that students in higher education have important differences from their non-enrolled peers and the general public [40]. Also, participants were informed about WGS by a video with demonstrated effectiveness [27], but our survey did not assess how engaged they were with the material or their absorption of knowledge. Furthermore, their responses are unlikely to match the perspectives of young adults who have had a detailed discussion about their decision-making process with the aid of a clinician. It has also been demonstrated that participant responses to hypothetical scenarios often indicate substantial interest in obtaining genetic testing, but studies sometimes observe lower uptake when these tests are offered to patients [41].

As the conversation about the implementation of WGS evolves, it is important to integrate the perspective young adults. Research exploring the impact of WGS in practice contexts will have greater applicability to clinical and policy decision making, as individuals who have experienced testing may have a more balanced view of its benefits and risks [42]. Finally, it is important for clinicians to recognize that young adults are likely to enter conversations about WGS with a positive outlook. Additional effort may need to be expended to ensure that this optimism is well-informed and results in a decision that is truly consistent with their patients’ values.

## Supporting information

S1 Document Survey questions. (PDF)

S1 Table Sources for survey items. (PDF)

S2 Table Sociodemographic factors correlated with interest in learning specific types of genomic information. (PDF)

S3 Table Attitudinal factors correlated with interest in learning specific types of genomic information. (PDF)
