# Supplementary material for: Preferences for the provision of whole genome sequencing services among young adults
Source: PLoS One. 2017 Mar 23;12(3):e0174131. doi: 10.1371/journal.pone.0174131 (PMC5363863; doi:10.1371/journal.pone.0174131)
Supplement: S1 Document — (PDF) [file pone.0174131.s001.pdf]

Supplemental Document 1: Survey Questions

Preferences for the Provision of Whole Genome Sequencing Services among Young Adults

Christopher H. Wade, PhD, MPH; Kailyn R. Elliott, BSN, RN

Author Note: This survey was offered online through the WebQ survey tool on the secure and proprietary University of Washington Catalyst System. Survey questions that are relevant to this publication are provided in the following document. Additional questions that are not addressed here can be found in another publication: Wade, C. H., & Elliott, K. (2016). Young Adults' Attitudes Toward Pediatric Whole Genome Sequencing. *Personalized Medicine* (In Press).

Are you 18 or 19 years-old?

	Logic destinations
○ Yes	⇨ Don't skip (default)
○ No	⇨ Unfortunately, we are only ...
No response	⇨ You have declined to partic...

By selecting "yes" below, you are providing your consent to be part of this study.

	Logic destinations
○ Yes, I give permission to be included in this study	⇨ Don't skip (default)
○ No, I do not want to be part of this study	⇨ You have declined to partic...
No response	⇨ End of Survey

Thanks for being willing to share your opinions in this survey!

Before you complete the survey, we want to give you some information about a new genetic technology using the video below, "Whole Genome Sequencing and You."

[Embedded Video: https://www.youtube.com/watch?v=IXamRS85hXU]

It is important to watch this video if you don't know a lot about the whole genome sequencing. It will help you to answer the survey questions accurately. If you are already

an expert in genomics, you may not need to watch it.

If you are on a mobile device or are having difficulty viewing the embedded video, you can also watch it in a separate window by clicking here or pasting https://www.youtube.com/watch?v=IXamRS85hXU into your address bar.

INSTRUCTIONS:

Please try to answer all of the questions in this survey to the best of your ability. If none of the options fit your exact opinion, please select the option that is closest to your opinion.

If you feel uncomfortable answering a question, you may select "Decline to Answer." We would really appreciate it if you try to answer, though. If you select "Decline to Answer" often, we may not be able to use *any* of your responses in the study.

First, we would like to learn a little bit about your background:

What is your age?

- ○ 18-years-old
- ○ 19-years-old
- ○ Decline to answer

Are you male or female?

- ○ Male
- ○ Female
- ○ Decline to answer

Are you of Hispanic or Latino origin?

- ○ Yes
- ○ No
- ○ Decline to answer

Regardless of the previous question, please mark the following box(es) to indicate what you consider your race to be. What race(s) do you consider yourself? (You may select more than one)

- ☐ African American or Black
- ☐ Alaska Native or American Indian
- ☐ Asian or Asian American

- White or Caucasian
- Native Hawaiian or other Pacific Islander
- Decline to answer
- Other:

What is your parents' approximate annual household income from all sources?

- Less than $25,000
- $25,001 to $50,000
- $50,001 to $75,000
- Over $75,000
- Decline to answer

In general, would you say your health is...

- Excellent
- Very good
- Good
- Fair
- Poor
- Decline to answer

We would like to ask you a few general questions about genetics and health

How important is it to you to learn more about how your **health habits** (like diet and exercise) affect your chance of getting certain health conditions?

- 1 - Not at all important
- 2
- 3
- 4
- 5
- 6
- 7 - Very important
- Decline to answer

How important is it to you to learn more about how your **genes**, that is the characteristics that are passed from one generation to the next, affect your chance of getting certain health conditions?

- 1 - Not at all important

- 2
- 3
- 4
- 5
- 6
- 7 - Very important
- Decline to answer

On a typical day, how motivated are you to do things that improve your health?

- 1 - Not at all motivated
- 2
- 3
- 4
- 5
- 6
- 7 - Very motivated
- Decline to answer

Does anyone close to you have a genetic disorder that influences their health?

- Yes
- No
- Decline to answer

In the future, you may have the opportunity to get whole genome sequencing. This could give you information about your chances of developing many different health conditions and traits.

Imagine that you had whole genome sequencing. How **interested** would you be in **learning** the following types of information?

Information Type A: Genomic information could show that a health condition is **very likely** to develop. However, you **can** take action to decrease your chances of the condition occurring.
Example: *People with Long QT Syndrome are at very high risk for having a sudden heart attack. However, if they know they have Long QT Syndrome, they can greatly decrease their chances of having a heart attack by taking medication and avoiding strenuous exercise.*
Would you want to know about this kind of information?

- 1 - Not at all interested
- 2
- 3
- 4
- 5
- 6
- 7 - Very interested
- Decline to answer

Information Type B: Genomic information could show that a health condition is **very likely** to develop. Unfortunately, you **can not** take action to decrease your chances of the condition occurring.
Example: *People with a certain genetic risk are very likely to develop Alzheimer's Disease, which is a fatal neurological condition that typically occurs later in life. There is no effective way to prevent this disease from occurring.*
Would you want to know about this kind of information?

- 1 - Not at all interested
- 2
- 3
- 4
- 5
- 6
- 7 - Very interested
- Decline to answer

Information Type C: Genomic information could show that your risk for a health condition is **slightly** or **moderately** increased. However, you **can** take action to decrease your chances that the condition will occur.
Example: *Sequencing could show that a person is two times more likely to have Type 2 Diabetes than the average person. Even though their risk is increased, there is still a good chance that they would not get Type 2 Diabetes. However, if they were motivated, they could change their diet and exercise behaviors to decrease their overall risk for diabetes.*
Would you want to know about this kind of information?

- 1 - Not at all interested
- 2
- 3
- 4
- 5
- 6
- 7 - Very interested

- Decline to answer

Information Type D: Genomic information could tell you about your **likely response to different types of medications**
Example: *Certain genetic information could show that a person would respond better to one type of cancer medication than another type. It could also improve a clinicians ability to select the right medication dose to decrease possible side effects.*
Would you want to know about this kind of information?

- 1 - Not at all interested
- 2
- 3
- 4
- 5
- 6
- 7 - Very interested
- Decline to answer

Information Type E: Genomic information could show that you are at greater **risk for having a child** with a rare genetic condition.
Example: *A person could find out that they are slightly more likely to have a child with Cystic Fibrosis (or much more likely if they know their partner also has a mutation). Children with Cystic Fibrosis have serious health difficulties, particularly with their ability to breathe.*
Would you want to know about this kind of information?

- 1 - Not at all interested
- 2
- 3
- 4
- 5
- 6
- 7 - Very interested
- Decline to answer

Information Type F: Genomic information could reveal that you are more likely to have **traits that are not necessarily good or bad** for your health.
Example: *A person could learn about their probability of having a certain appearance, memory capacity, or athletic ability.*
Would you want to know about this kind of information?

- 1 - Not at all interested
- 2

- 3
- 4
- 5
- 6
- 7 - Very interested
- Decline to answer

Information Type G: Genomic information could suggest your likely ancestral heritage
Example: *A person could learn about which countries or regions their ancestors probably lived in.*
Would you want to know about this kind of information?

- 1 - Not at all interested
- 2
- 3
- 4
- 5
- 6
- 7 - Very interested
- Decline to answer

Imagine that you had whole genome sequencing. **Would you want to know** if you had **increased chances** of developing the following health conditions or traits?

Rows

Long QT Syndrome (sudden heart attack)
Alzheimer's disease
Heart disease
Diabetes
Obesity
Prostate cancer
Breast cancer
Lung cancer
Depression
Flu virus infection risk
Alcoholism
Having a child with Cystic Fibrosis
Responding well to a medication
Athletic ability
Memory traits
Appearance traits
Having ancestors that lived in a specific global location

- Yes

- No
- Decline to answer

Please tell us the extent to which you agree or disagree with each of the following statements about getting your whole genome sequenced:

Rows

It would give me important information
It would make me uneasy
It would be an invasion of my privacy
It would help me stay healthy
It would give me useless information
It would help me to pay more attention to my health
It would help me prevent health problems
It is not important
It might tell me something I don't want to know
It would help me put my mind at ease

- 1 - Strongly disagree
- 2
- 3
- 4
- 5
- 6
- 7 - Strongly agree
- Decline to answer

How likely is it that knowing the results from whole genome sequencing for yourself would lead to any changes in your behavior?

- Not at all likely
- Not very likely
- Not sure
- Quite likely
- Very likely
- Decline to answer

Imagine that you had your whole genome sequenced and learned that you had a **higher chance** of developing a serious health condition. Do you think you would be able to **handle the emotional impact** of this information?

- 1 - No, I would not
- 2

- 3
- 4
- 5
- 6
- 7 - Yes, I would
- Decline to answer

We would also like to learn a little bit about your **overall opinions** toward whole genome sequencing

How willing would you be to have your whole genome sequenced?

- 1 - Not at all willing
- 2
- 3
- 4
- 5
- 6
- 7 - Very willing
- Decline to answer

How much would you be willing to pay to have you whole genome sequenced?

- I wouldn't want whole genome sequencing
- I would want whole genome sequencing but wouldn't pay for it ($0)
- $1 to $50
- $51 to $200
- $201 to $500
- $501 to $1000
- $1001 to $3000
- Over $3000
- Decline to answer

We would like to ask you some questions now about what types of choice are important to you when using whole genome sequencing information in your healthcare.

Imagine that your healthcare provider decided what information to tell you about your whole genome sequence. Assume your provider did their best to **only tell you** information that he or she thought could **improve your health**. **You do not get to**

decide what types of information you are told.

In this scenario, how **willing** would you be to undergo whole genome sequencing?

- 1 - Not at all willing
- 2
- 3
- 4
- 5
- 6
- 7 - Very willing
- Decline to answer

You may be given a choice about what types of information you learn about your whole genome sequence. How **important is it to you to be able to choose** what types of information you are told?

- 1 - Not at all important
- 2
- 3
- 4
- 5
- 6
- 7 - Very important
- I wouldn't want sequencing
- Decline to answer

Which of the following statements **best match** your opinion about **who should decide** what information you learn from whole genome sequencing?

- I want my healthcare provider to make the decisions about what information is important for me to know
- I want my healthcare provider to pick out the results that he or she sees as most important. Then I want to choose which types of information my healthcare provider tells me
- I want to be able to choose general categories of test results that I want to know about (such a very likely health risks, drug responses, or traits). Then I want my healthcare provider to tell me all of the results in the categories that I pick
- I want to be able to choose among each separate health condition, drug response, or trait (such as diabetes, depression, athletic ability, etc.). Then I want my healthcare provider to tell me all the results for the specific conditions that I pick
- I wouldn't want sequencing
- Decline to answer

Finally, we would like to hear your thoughts on whole genome sequencing **in your own words**. This often gives us a much better understanding of your perspective than multiple-choice style questions.

Imagine you had to decide whether or not to get whole genome sequencing. **What factors** would be most important to your decision?

Imagine you got whole genome sequencing. How do you think that you would **use the information** that you learned?
