# Supplementary material for: Preferences for the provision of whole genome sequencing services among young adults
Source: PLoS One. 2017 Mar 23;12(3):e0174131. doi: 10.1371/journal.pone.0174131 (PMC5363863; doi:10.1371/journal.pone.0174131)
Supplement: S1 Table — (PDF) [file pone.0174131.s002.pdf]

Supplemental Table 1: Sources for Survey Items

Preferences for the Provision of Whole Genome Sequencing Services among Young Adults

Christopher H. Wade, PhD, MPH; Kailyn R. Elliott, BSN, RN

Measures are provided in the order presented in the survey, which can be reviewed in **Supplemental Document 1**.

Survey Measure	Source
Age	Adapted from HINTS 4[1]
Gender	HINTS 4[1]
Ethnicity	University of Washington Bothell Institutional Data Collection Tool
Race	University of Washington Bothell Institutional Data Collection Tool
First generation college student (defined as neither parent having attended college)	University of Washington Bothell Institutional Data Collection Tool
Parent's household income	Adapted from BRFSS[2]
Health status	HINTS 4[1]
Importance behavior	Tercyak et. al. (2011)[3]
Importance genes	Tercyak et. al. (2011)[3]
Motivation to improve health	New
Know a person with a genetic disorder	New
Interest in types of WGS information	New
Interest in specific health conditions & traits	New
Decisional balance scale	Adapted from Tercyak et. al. (2011)[3]
WGS will change behavior	Sanderson et. al. (2013)[4]
Ability to handle emotions	New
Willingness to get WGS	Adapted from Tercyak et. al. (2011)[3]
Willingness to pay for WGS	New
Willingness to get WGS – No choice scenario	New
Importance of choice	New
Preferred specificity of choice	New

References

1. U.S. Department of Health and Human Services. Health Information National Trends Survey 4 (HINTS 4) Cycle 3 Methodology Report. 2014.

2. Centers for Disease Control and Prevention (CDC). Behavioral Risk Factor Surveillance System Survey Questionnaire. 2013.

3. Tercyak KP, Hensley Alford S, Emmons KM, Lipkus IM, Wilfond BS, McBride CM. Parents' attitudes toward pediatric genetic testing for common disease risk. Pediatrics 2011 May;127(5):e1288-95.

4. Sanderson SC, Linderman MD, Kasarskis A, Bashir A, Diaz GA, Mahajan MC, et al. Informed decision-making among students analyzing their personal genomes on a whole genome sequencing course: a longitudinal cohort study. Genome Med 2013 Dec 30;5(12):113.
