# Supplementary material for: Preferences for the provision of whole genome sequencing services among young adults
Source: PLoS One. 2017 Mar 23;12(3):e0174131. doi: 10.1371/journal.pone.0174131 (PMC5363863; doi:10.1371/journal.pone.0174131)
Supplement: S2 Table — (PDF) [file pone.0174131.s003.pdf]

Supplementary Table 2: Sociodemographic Factors Correlated with Interest in Learning Specific Types of Genomic Information

Interest in learning:	Hispanic or Latino, *r* (*P*)[ab]	African American, *r* (*P*)[ab]	Asian or Asian American, *r* (*P*)[ab]	Caucasian, *r* (*P*)[ab]	Parents' Annual Household Income, *r* (*P*)[ab]
**Predictive information about preventable health conditions**	.09 (.30)	-.19 (.02)*	.01 (.96)	.15 (.09)	.13 (.15)
**Predictive information about non-preventable health conditions**	.05 (.57)	.12 (.15)	.04 (.61)	-.03 (.74)	.09 (.35)
**Slight or moderate susceptibility to health conditions**	.11 (.19)	-.04 (.69)	.07 (.43)	.05 (.56)	.183 (.05)*
**Pharmacogenomic information**	.12 (.14)	.06 (.46)	-.12 (.15)	.14 (.11)	.24 (<.01)*
**Carrier status**	-.035 (.68)	.05 (.59)	.07 (.41)	.036 (.67)	.151 (.11)
**Non-health related traits**	.11 (.17)	.13 (.14)	-.21 (.01)*	.20 (.02)*	.06 (.52)
**Ancestry**	.22 (<.01)*	-.03 (.76)	-.15 (.08)	.134 (.11)	.02 (.86)

[a] Asterisk highlights correlations of $P \leq .05$.

[b] Inclusion in this table required that a sociodemographic factor have a significant relationship with at least one type of genomic information.
