# Supplementary material for: Preferences for the provision of whole genome sequencing services among young adults
Source: PLoS One. 2017 Mar 23;12(3):e0174131. doi: 10.1371/journal.pone.0174131 (PMC5363863; doi:10.1371/journal.pone.0174131)
Supplement: S3 Table — (PDF) [file pone.0174131.s004.pdf]

Supplementary Table 3: Attitudinal Factors Correlated with Interest in Learning Specific Types of Genomic Information

Interest in learning:	Motivation to take action to improve health, r (P)[ab]	Knowing WGS results would lead to change in behavior, r (P)[ab]	Able to handle emotions if WGS revealed risk for a serious health condition, r (P)[ab]	Willingness to pay for WGS, r (P)[ab]	Importance of being able to choose what types of WGS information is reported, r (P)[ab]
**Predictive information about preventable health conditions**	.09 (.27)	.252 (<.01)*	.14 (.10)	.10 (.23)	.18 (.03)*
**Predictive information about non-preventable health conditions**	.09 (.32)	.05 (.54)	.25 (<.01)*	.08 (.36)	.10 (.26)
**Slight or moderate susceptibility to health conditions**	.08 (.37)	.21 (.01)*	.27 (<.01)*	.09 (.29)	.15 (.08)
**Pharmacogenomic information**	.24 (<.01)*	.23 (.01)*	.23 (.01)*	.23 (.01)*	.27 (<.01)*
**Carrier status**	.00 (1.00)	.21 (.01)*	.28 (<.01)*	.20 (.02)*	.18 (.04)*
**Non-health related traits**	.15 (.08)	.02 (.84)	.07 (.38)	-.01 (.90)	.02 (.81)
**Ancestry**	.13 (.11)	.00 (.97)	.11 (.19)	-.08 (.37)	.20 (.02)*

[a] Asterisk highlights correlations of $P \leq .05$.
[b] Inclusion in this table required that an attitudinal factor have a significant relationship with at least one type of genomic information.
